# Characterization of Vasoreactivity in a Semi-Arboreal Snake, the Tokara Habu (*Protobothrops tokarensis*)

**DOI:** 10.3390/ani13233629

**Published:** 2023-11-23

**Authors:** Tomoki Ootawa, Siyuan Wu, Ryoya Sekio, Henry Smith, Md. Zahorul Islam, Ha Thi Thanh Nguyen, Yasuhiro Uno, Mitsuya Shiraishi, Atsushi Miyamoto

**Affiliations:** 1 Department of Basic Veterinary Science, Joint Graduate School of Veterinary Medicine, Kagoshima University, Kagoshima 890-0065, Japan; k2773273@kadai.jp (T.O.); k0460053@kadai.jp (S.W.); k5908476@kadai.jp (H.S.);; 2 Japan Wildlife Research Center, Tokyo 130-8606, Japan; 3 Department of Veterinary Pharmacology, Joint Faculty of Veterinary Medicine, Kagoshima University, Kagoshima 890-0065, Japan; 4 Department of Pharmacology, Faculty of Veterinary Science, Bangladesh Agricultural University, Mymensingh 2202, Bangladesh; 5 Department of Veterinary Pharmacology and Toxicology, Faculty of Veterinary Medicine, Vietnam National University of Agriculture, Hanoi 131000, Vietnam

**Keywords:** acetylcholine, adrenoceptors, environment, habitat, Tokara Islands, serotonin, snake, vasoreactivity

## Abstract

**Simple Summary:**

Snakes are reptiles that have evolved over a period of approximately 170 million years, adapting to life in different habitats, including water (rivers and oceans), on the ground, in trees, and underground. The distance from the heart to the brain is known to be shorter in arboreal snakes compared to terrestrial ones, indicating that differences in habitat may also affect vascular response. In this study, we attempted to characterize vasoreactivity in the Tokara habu, a semi-arboreal snake. The Tokara habu snakes demonstrated a number of relaxation responses to various vasoactive substances, and they showed complex vasoreactivity, a contrast to the simple vasoreactivity seen in terrestrial snakes. These results suggest that the Tokara habu’s distinctive, complex vasoreactivity may reflect adaptation to its semi-arboreal environment. Comparisons of vascular responses may be useful as a new approach to behavioral and ecological studies for species that are difficult to observe in the field.

**Abstract:**

Vasoreactivity is relatively well documented in terrestrial snakes but has previously been investigated in only one semi-arboreal snake species. Consequently, the extent to which vasoreactivity is common across snake taxa or varies by habitat is unclear. The Tokara habu (*Protobothrops tokarensis*) is a semi-arboreal snake endemic to only two small adjacent Japanese islands, and hence a useful species for further investigation of vasoreactivity. We evaluated responses to known vasoactive substances in thoracic aortas isolated from Tokara habu. Under resting tension, noradrenaline and angiotensin II induced concentration-dependent contraction, but acetylcholine, serotonin (5-hydroxytriptamine; 5-HT), and isoproterenol induced relaxation followed by contraction. Histamine and rattlesnake bradykinin had no effect. Experiments with receptor-specific antagonists suggest that M_1_ and M_3_ receptors are involved in the acetylcholine-induced response; 5-HT_1_, 5-HT_2_, and 5-HT_7_ receptors in the serotonin-induced response; and β_1_ and β_2_ adrenoceptors in isoproterenol-induced relaxation. This is the first report on such response patterns in snakes (including serotonin- and isoproterenol-induced relaxation). Nitric oxide may be involved in acetylcholine-induced relaxation but not in the responses to serotonin or isoproterenol. In contrast to the uniform vasoreactivity observed in terrestrial snakes, the vasoreactivity of semi-arboreal snakes may be governed by diverse regulatory mechanisms.

## 1. Introduction

Snakes have colonized an environmentally diverse range of niches since they first evolved between 174 and 163 million years ago [1]. Their adaptations to these habitats present an interesting contrast to those seen in many other vertebrates [2]. Examples of habitat-adapted taxa include arboreal primates, various terrestrial quadrupeds, and cetaceans among mammals; and tree-nesting birds of flight, terrestrial birds such as ostriches, and semi-aquatic penguins among avian species. Snakes are distinctive as a group of taxa because they are found in terrestrial, arboreal, subterrain, and aquatic environments, and many of their basic characteristics (including tissue organization) are common at the order level in Linnean classification—specifically for the suborder Serpentes—unlike birds and mammals, which tend to show such characteristics at the class level. Snakes are thus useful animals for understanding environmental adaptations to a range of habitats. However, the mechanisms underlying these adaptations remain largely unknown [3], and their elucidation would be of great benefit for evolutionary studies.

Snakes vary in size, but they share a common shape. Their cardiovascular systems thus all operate within a similar cranial–caudal arrangement but confront different habitat-related challenges in achieving systemic circulation. A major example of such a challenge is gravity, which has effects on terrestrial, marine, and arboreal species, generating different levels of hydrostatic pressure, which the circulation must overcome [3,4,5]. A useful way to understand how the circulation achieves this is to investigate vasoreactivity. This investigation will reveal how the vascular endothelium and smooth muscles react to a range of potentially vasoactive substances and what receptor populations they possess.

Vasoreactivity has been studied in the thoracic aorta in three terrestrial snakes [6,7,8,9], spanning the Natricinae, Elapidae, and Viperidae families. Common features across these reports included noradrenaline-induced contractions in two of three species [7,9], angiotensin II-induced contractions in two of three species [6,7], and acetylcholine-induced relaxation in one of three species [8]. Vasoreactivity has been investigated in only one semi-arboreal snake, the yellow rat snake (*Pantherophis obsoletus*) [10], which showed both some similarities and some differences with the terrestrial snakes. Broadly, this may suggest that basic vasoreactivity may be similar in species colonizing similar habitats. Further investigations in other semi-arboreal snakes are required to establish whether snakes in this habitat exhibit a similar pattern of vasoreactivity.

A semi-arboreal species of potential interest for physiologists is the Tokara habu (*Protobothrops tokarensis*), a mildly venomous snake of the Viperidae family, which is found in only two small, adjacent islands in the southwestern Japanese Archipelago (Takarajima and Kodakarajima) [11]. This insular species is phylogenetically very close to the Habu (*Protobothrops flavoviridis)* [12], a terrestrial species inhabiting other parts of this Japanese island chain [13]. However, the Tokara habu is a distinct species dwelling in a semi-arboreal habitat on islands now separated from the neighboring Amami Islands, where their close relatives, the Habu, largely dwell in a terrestrial habitat. The Tokara habu is thus very interesting from the perspective of its physiological relationship to the environment [11].

Accordingly, in the present study, we aimed to characterize thoracic aortic vasoreactivity in the Tokara habu, as a representative semi-arboreal snake species, by applying a range of vasoactive substances (including noradrenaline, angiotensin II, and acetylcholine) to aortic rings harvested from snake carcasses in Kodakarajima. Furthermore, we conducted a field survey to verify the semi-arboreal behavior of the Tokara habu.

## 2. Materials and Methods

### 2.1. Sampling Location

**Figure 1:**
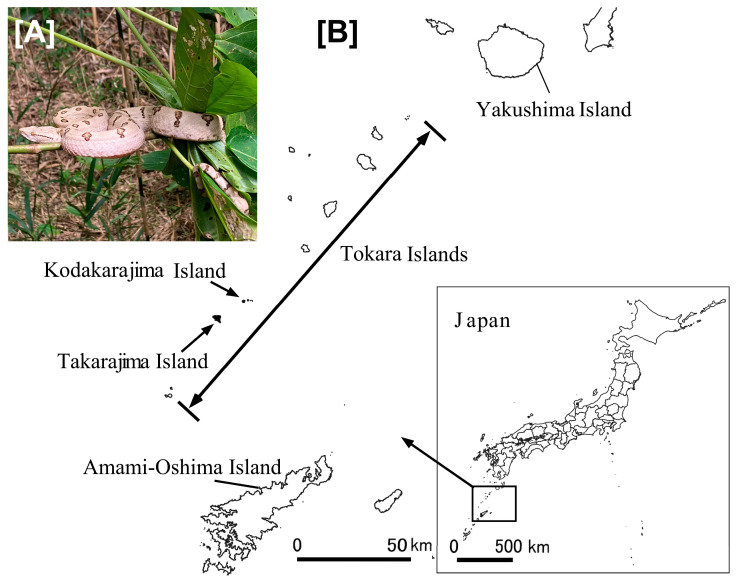
(**A**) A Tokara habu (*Protobothrops tokarensis*) on a tree. (**B**) Locations of Takarajima and Kodakarajima within the Tokara Islands, Japan. This figure was created by processing relevant data from the Geospatial Information Authority of Japan (GSI) tiles [14].

We obtained the Tokara habu carcasses from the island of Kodakarajima (latitude: 29°22′ E; longitude: 129°32′ N), which belongs to the Tokara Archipelago, Kagoshima Prefecture, Japan. The Tokara habu are 0.6–1.1 m in length and have a snout-vent length of less than 0.9 m [11]. The snakes’ habitat consists of secondary forests (where they reportedly often climb trees), shrublands, and grasslands [11]. A representative Tokara habu snake and the sampling location are shown in Figure 1.

### 2.2. Ethics Approval

Before the collection of snake carcasses, we submitted an experimental proposal to the Residential Affairs Division of Toshima Village Office, from which approval for the experiments (Approval Nos. 2260, 2930, and 2163) was obtained. All experiments were performed in accordance with the Guidelines for Animal Experiments of Kagoshima University. Since this experiment used arteries isolated from previously exterminated snakes, individual ethical and animal experimental approval was not required from Kagoshima University. AAALAC International has fully accredited our animal experimental facilities and programs since 2017.

### 2.3. Vascular Tissue Sampling and Tissue Preparation

We harvested tissue from a total of 22 Tokara habu snakes (mature adults), which had been captured on Kodakarajima and euthanized as noxious animals by Toshima Village office personnel with carbon dioxide gas inhalation in March 2020, September 2021, or October 2022.

The carcasses (males: *n* = 15, females: *n* = 7, body weight: 159.95 ± 37.6 g; snout-vent length: 0.75 ± 0.01 m) were selected randomly from euthanized adults confirmed macroscopically to have been in good body condition and free of any visible signs of injury, parasites, or disease. Vascular tissue was collected from each selected carcass within 60 min of completion of the euthanasia procedures.

### 2.4. Substances and Reagents

We used the following reagents at the final concentrations shown: noradrenaline (10^−9^–10^−5^ M), phentolamine mesylate (10^−6^ M), isoproterenol (10^−9^–10^−5^ M), propranolol (10^−7^ M), histamine hydrochloride (10^−9^–10^−5^ M), serotonin (5-hydroxytriptamine; 5-HT; 10^−9^−10^−5^ M), ketanserin tartrate (10^−6^ M), methiothepin maleate (10^−8^ M), SB269970 (I-3-(2-(2-(4-methylpiperidin-1-yl)-ethyl)pyrrolidine-1-sulphonyl) phenol hydrochloride), rattlesnake bradykinin ((Val^1^,Thr^6^)-bradykinin) (10^−9^–10^−5^ M), which was synthesized by Shimadzu Co. (Kyoto, Japan); atenolol (10^−6^ M, LKT Laboratories, Tokyo, Japan), butoxamine hydrochloride (10^−6^ M, Sigma-Aldrich, St. Louis, MO, USA), N^ω^-nitro-L-arginine (L-NNA; 10^−4^ M), sodium nitroprusside (SNP; 10^−4^ M, Nacalai Tesque, Kyoto, Japan), acetylcholine (10^−9^–10^−5^ M, Daiichi Sankyo, Tokyo, Japan), pirenzepine dihydrochloride (10^−6^ M; Santa Cruz Biotechnology, Santa Cruz, CA, USA), methoctramine hydrate (10^−6^ M), hexahydro-sila-difenidol hydrochloride, p-fluoroanalog (pfHHSiD; 10^−6^ M), and angiotensin II (10^−9^–10^−5^ M). All reagents were employed in accordance with the relevant manufacturer’s guidelines. SB266970 was initially dissolved in dimethyl sulfoxide (DMSO) at 1 mM. The final DMSO concentration was 0.1% (*v*/*v*). All other drugs were dissolved in water. Each selected agent had previously been demonstrated to be an agonist in vertebrate taxa (including reptilian taxa) [15,16,17].

### 2.5. Functional Study

Vasoreactivity was investigated in accordance with a previously published method [18] with some modifications. Briefly, five rings, each approximately 3 mm long, were cut from the thoracic aorta of each exterminated snake. Each ring was mounted horizontally between two L-shaped stainless-steel holders, with one part fixed to an isometric force transducer, and immersed in a 5 mL water-jacketed organ bath (UMTB-a, Unique Medical Co., Ltd., Tokyo, Japan) containing oxygenated salt solution at 28.0 ± 0.5 °C (pH 7.4). The resting tension in each thoracic aorta was 2.4 mN.

Vascular tension strength was calculated based on percentage values for contraction (the contraction induced by 60 mM KCl was taken as 100%) and for relaxation (the relaxation induced by 10^−4^ M sodium nitroprusside was taken as 100%).

### 2.6. Nitric Oxide Quantification Using Fluorescence

Nitric oxide (NO) was quantified using aorta specimens prepared in accordance with a previously described method [19], with some modifications. Briefly, each thoracic aorta (*n* = 5 exterminated snakes; total: 1.20 ± 0.05 mg wet tissue per specimen) was cut into 4 mm segments and immersed in a 500 µL tube containing oxygenated physiological saline at 28 °C (pH 7.4). The segments were then treated with serotonin (10^−6^ M), acetylcholine (10^−7^ M), or isoproterenol (10^−6^ M) to achieve the relevant experimental condition. The involvement of endothelial NO was also investigated with comparator treatments involving pretreatment with L-NNA and incubation for 1 h.

### 2.7. Statistical Analysis

Acquired data included the pEC_50_ values and E_max_ for each potentially vasoactive substance. The pEC_50_ value indicates the sensitivity of the substance to the corresponding receptor, and E_max_ indicates the strength of its effect on the vascular response. Higher pEC_50_ values thus indicate higher sensitivity to the binding receptor, and higher E_max_ indicates a greater impact on the vascular endothelial response.

Results are expressed as means ± SEM. Statistical analyses were performed using Student’s *t*-test for comparisons between two groups or the Bonferroni test after one-way analysis of variance (Stat View J-4.5, Abacus Concepts Inc., Berkeley, CA, USA) for comparisons between three or more groups. Results were regarded as significant when the probability of rejecting a true null hypothesis was equal to or less than 5%.

### 2.8. Field Research

To investigate the Tokara habu’s semi-arboreal behavior (how often and how high they climb), we carried out field observations to generate reference data. The field research was conducted over the entire area of Kodakarajima (0.98 km^2^), on two designated dates, one in September 2021 and one in October 2022. The field research on each designated date was carried out by a single observer, for an approximately seven-hour observation period that included periods of daylight and natural darkness. Observations in the dark period were continued beyond midnight, but for recording purposes, the field research day was regarded as that on which the observations started. For each Tokara habu identified in these searches, we recorded the spatial position (on the ground or in a tree) and the height from the ground (in meters).

## 3. Results

### 3.1. Responsiveness to Noradrenaline, Angiotensin II, Histamine, Rattlesnake Bradykinin, Isoproterenol, Serotonin, and Acetylcholine

**Figure 2:**
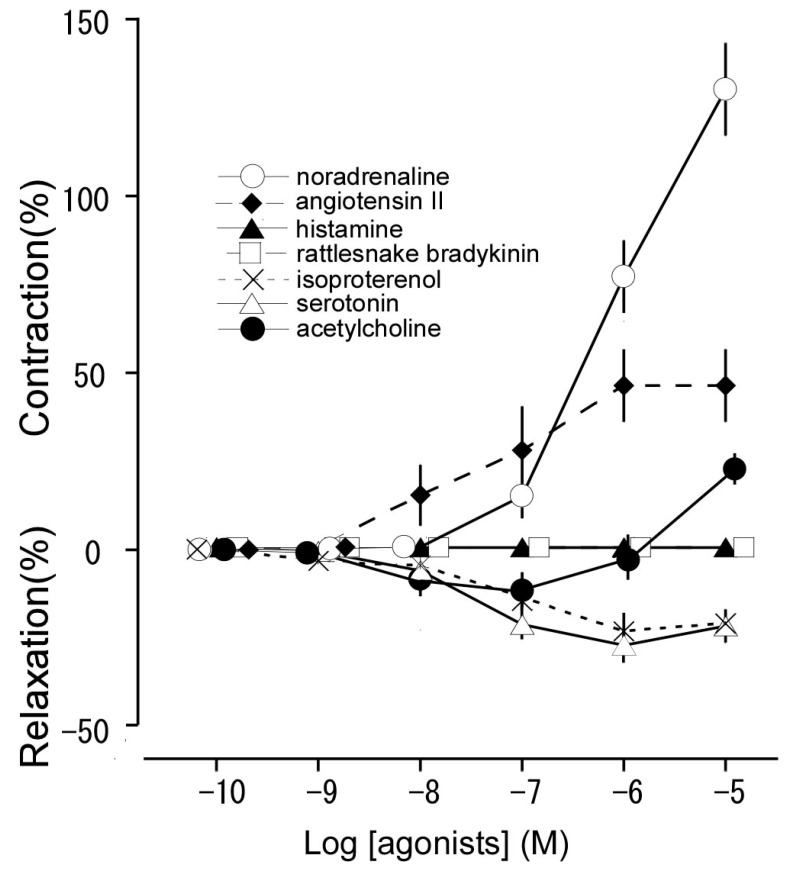
Responsiveness to noradrenaline (○), angiotensin II (♦), histamine (▲), rattlesnake bradykinin (□), isoproterenol (×), serotonin (Δ), and acetylcholine (●) in isolated Tokara habu thoracic aortas under resting tension. The contraction induced by 60 mM KCl was taken as 100%. The relaxation induced by sodium nitroprusside (10^−4^ M) was taken as 100%. Each point represents the mean ± SEM for 12 snakes.

We generated concentration-response curves for noradrenaline, angiotensin II, histamine, rattlesnake bradykinin, isoproterenol, serotonin, and acetylcholine based on data from thoracic aortas isolated from the Tokara habu carcasses (Figure 2). Noradrenaline and angiotensin II induced contraction in a concentration-dependent manner. Isoproterenol, serotonin, and acetylcholine induced relaxation at a low concentration (10^−9^ M–10^−7^ or 10^−6^ M), followed by contraction at a high concentration (10^−6^ M–10^−5^ or only 10^−5^ M), in resting tension. Histamine and rattlesnake bradykinin had no effect on the resting tension or the precontracted condition induced with noradrenaline (10^−6^ M). The maximum contractile responses for noradrenaline and angiotensin II, the maximal relaxation responses for isoproterenol, serotonin, and acetylcholine, and the pEC_50_ (indicating sensitivity for the receptor) and E_max_ (indicating response strength) values for all vasoactive substances are shown in Table 1.

### 3.2. Effect of Phentolamine on Noradrenaline-Induced Contraction

**Figure 3:**
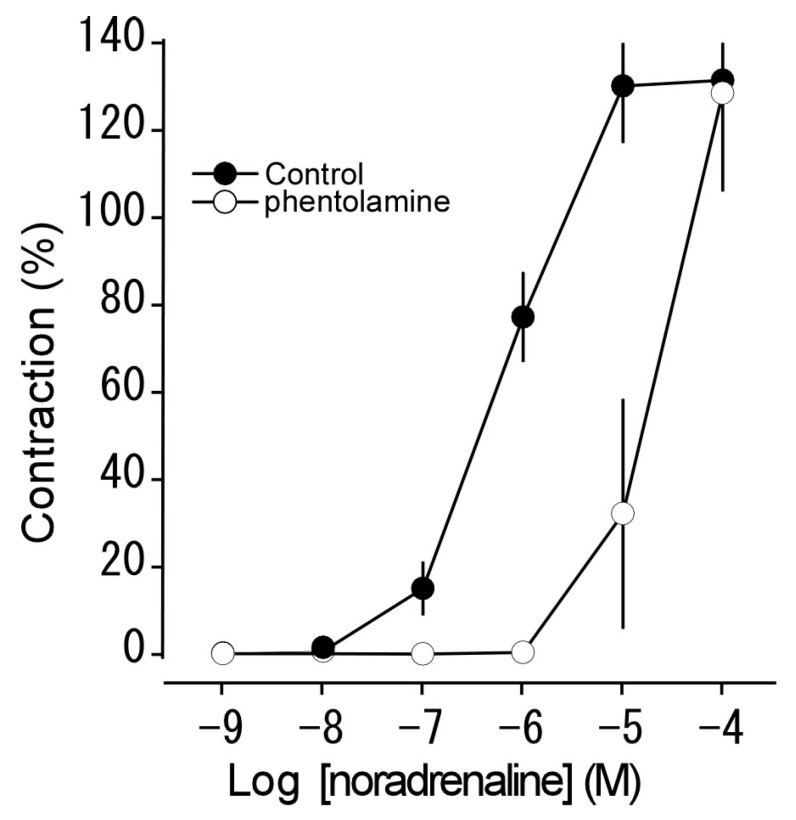
Effect of phentolamine (○: a non-selective α adrenoceptor antagonist, 10^−6^ M) on noradrenaline-induced contraction (●: Control) in isolated thoracic aortas. The contraction induced by 60 mM KCl was taken as 100%. Each point represents the mean ± SEM for 6 snakes.

We examined the effect of phentolamine (10^−6^ M, a non-selective α adrenoceptor antagonist) on noradrenaline-induced contraction. Phentolamine shifted the concentration-response curve for noradrenaline to the right in parallel (Figure 3).

### 3.3. Effects of β Adrenoceptor Antagonists on Isoproterenol-Induced Relaxation

**Figure 4:**
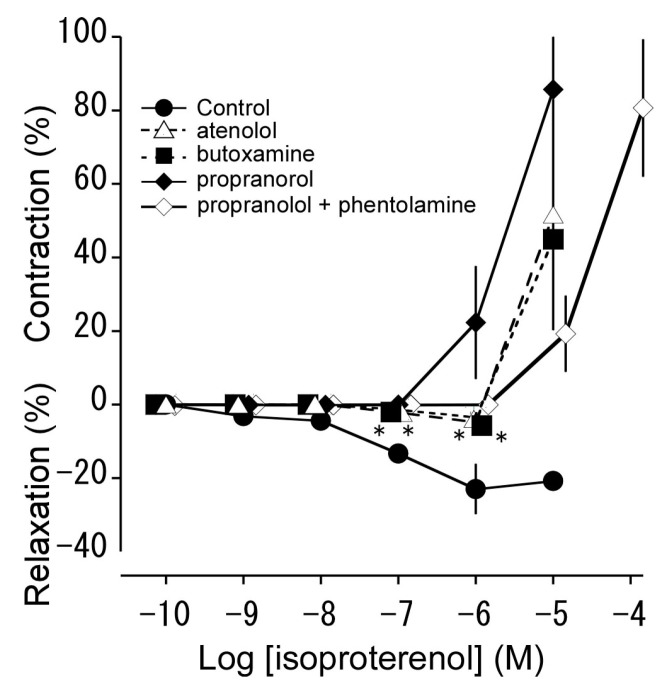
Effects of propranolol (♦: 10^−7^ M, a β_1_ and β_2_ adrenoceptor antagonist), atenolol (Δ: 10^−6^ M, a selective β_1_ receptor antagonist), butoxamine (■: 10^−6^ M, a selective β_2_ receptor antagonist), and propranolol (10^−6^ M) + phentolamine (10^−6^ M) on isoproterenol-induced relaxation and contraction (●: Control) in isolated thoracic aortas. The contraction induced by 60 mM KCl was taken as 100%. The relaxation induced by sodium nitroprusside (10^−4^ M) was taken as 100%. Each point represents the mean ± SEM for 6 snakes. (* *p* < 0.05 vs. Control).

We examined the effects of propranolol (10^−7^ M, a non-selective β adrenoceptor antagonist), atenolol (10^−6^ M, a β_1_ adrenoceptor antagonist), and butoxamine (10^−6^ M, a β_2_ adrenoceptor antagonist) on isoproterenol-induced relaxation in resting tension (Figure 4). Propranolol completely abolished isoproterenol-induced relaxation. Atenolol and butoxamine also had effects in the same direction, changing isoproterenol-induced relaxation to contraction, but their effects were less strong than that of propranolol. Phentolamine shifted the isoproterenol-induced contraction to the right in parallel in the presence of propranolol.

### 3.4. Effects of Serotonin Receptor Antagonists and L-NNA on Serotonin-Induced Responses 

**Figure 5:**
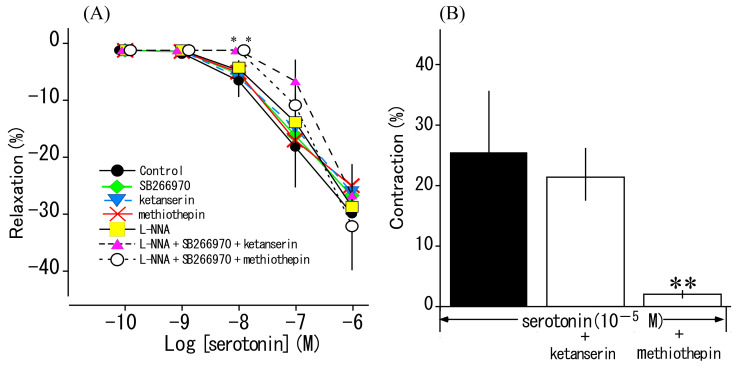
Effects of SB269970, methiothepin, ketanserin, and N^ω^-nitro-L-arginine (L-NNA) on serotonin-induced relaxation or contraction. (**A**): Effect of SB269970 (◊: 10^−7^ M, *n* = 6), ketanserin (∇: 10^−6^ M, *n* = 6), methiothepin (×: 10^−8^ M, *n* = 6), L-NNA (□: 10^−4^ M, *n* = 6), L-NNA (10^−4^ M) + SB269970 (10^−7^ M) + methiothepin (10^−8^ M) (Δ, *n* = 4), and L-NNA (10^−4^ M) + SB269970 (10^−7^ M) + ketanserin (10^−6^ M) (○, *n* = 4) on serotonin-induced relaxation (●: Control, *n* = 12). The relaxation induced by 10^−4^ M sodium nitroprusside was taken as 100%. Each point represents the mean ± SEM. * *p* < 0.05 compared with the value of Control. (**B**): Effects of methiothepin (10^−8^ M) and ketanserin (10^−6^ M) on serotonin-induced contraction (black: 10^−5^ M, Control). The contraction induced by 60 mM KCl was taken as 100%. Each bar represents the mean ± SEM for 6 snakes. ** *p* < 0.01 compared with the value of Control.

We examined the effects of L-NNA (a NO synthase inhibitor), methiothepin (a 5-HT_1_ and 5-HT_2_ receptor antagonist), ketanserin (a 5-HT_2_ receptor antagonist), and SB269770 (a 5-HT_7_ receptor antagonist) on serotonin-induced relaxation. No antagonist or inhibitor significantly reduced serotonin-induced relaxation. We then examined the effect of these substances when applied in combinations, and found that a combination of L-NNA, SB269970, and methiothepin or ketanserin abolished serotonin-induced relaxation at 10^−9^ and 10^−8^ M but had no significant effect at 10^−7^ or 10^−6^ M (Figure 5A). Serotonin had induced contraction at 10^−5^ M (indicated with the Δ symbol in Figure 2); therefore, we investigated the effects of methiothepin and ketanserin on this contraction, and the results are shown in Figure 5B. Methiothepin, but not ketanserin, significantly inhibited serotonin (10^−5^ M)-induced contraction (*p* < 0.01).

### 3.5. Effects of Muscarine Receptor Antagonists on Acetylcholine-Induced Relaxation

**Figure 6:**
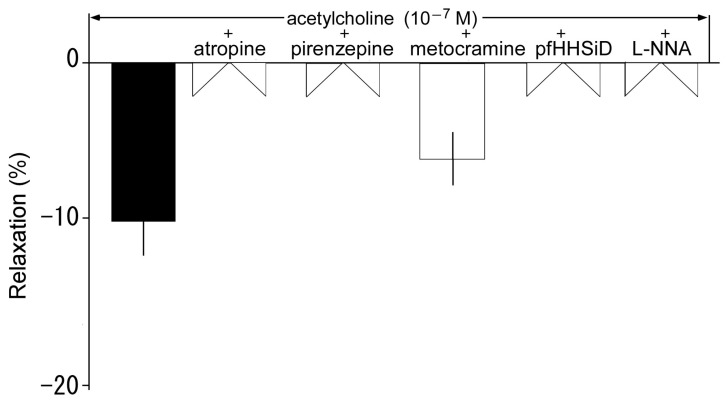
Effects of atropine (10^−6^ M, a non-selective muscarine receptor antagonist), pirenzepine (10^−6^ M, an M_1_ receptor antagonist), methoctramine (10^−6^ M, an M_2_ receptor antagonist), hexahydro-sila-difenidol hydrochloride, p-fluoroanalog (pfHHSiD) (10^−6^ M, an M_3_ receptor antagonist), and N^ω^-nitro-L-arginine (L-NNA) (10^−4^ M) on acetylcholine-induced relaxation (black: 10^−7^ M) in thoracic aortas in resting tension. The relaxation induced by sodium nitroprusside (10^−4^ M) was taken as 100%. Each bar represents the mean ± SEM for 6 snakes.

Acetylcholine induced relaxation at a low concentration of 10^−9^–10^−7^ M. To clarify the receptor subtype involved in this relaxation and the involvement of NO, we then applied several muscarinic (M) receptor antagonists and L-NNA. Relaxation induced by acetylcholine (10^−7^ M) was abolished by atropine (a non-selective M receptor antagonist), L-NNA, pirenzepine (an M_1_ receptor antagonist), and pfHHSiD (an M_3_ receptor antagonist) but not by methoctramine (an M_2_ receptor antagonist), as shown in Figure 6.

### 3.6. Effects of Muscarine Receptor Antagonists on Acetylcholine-Induced Contraction in the Presence of L-NNA

**Figure 7:**
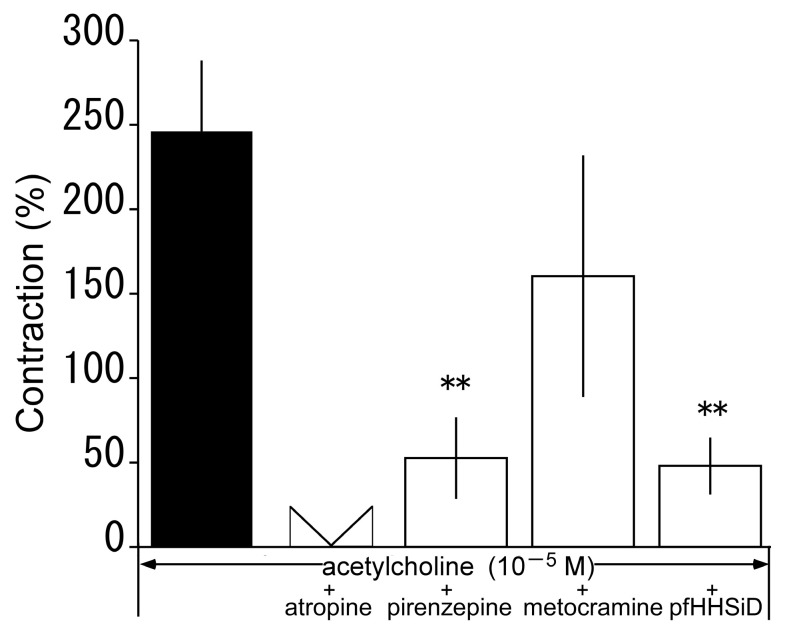
Effects of atropine (10^−6^ M, a non-selective M receptor antagonist), pirenzepine (10^−6^ M, an M_1_ receptor antagonist), methoctramine (10^−6^ M, an M_2_ receptor antagonist), and hexahydro-sila-difenidol hydrochloride, p-fluoroanalog (pfHHSiD) (10^−6^ M, an M_3_ receptor antagonist) on acetylcholine-induced contraction (black: 10^−5^ M, Control) in Tokara habu thoracic aortas in the presence of N^ω^-nitro-L-arginine (L-NNA) (10^−4^ M). The contraction induced by 60 mM KCl was taken as 100%. Each bar represents the mean ± SEM for 5 snakes. ** *p* < 0.01 compared with the value of Control.

Acetylcholine induced contraction at high concentrations of 10^−6^ and 10^−5^ M (● in Figure 2). Since acetylcholine-induced relaxation was abolished by L-NNA (Figure 6), we examined the effect of M receptor antagonists on acetylcholine-induced contraction in the presence of L-NNA (Figure 7). The treatment with atropine abolished acetylcholine (10^−5^ M)-induced contractions completely. The treatments with pirenzepine (10^−6^ M) and pfHHSiD (10^−6^ M) significantly inhibited acetylcholine-induced contractions (*p* < 0.01), whereas methoctramine (10^−6^ M) had no significant effect.

### 3.7. Nitric Oxide Production by Serotonin, Acetylcholine, and Isoproterenol

**Figure 8:**
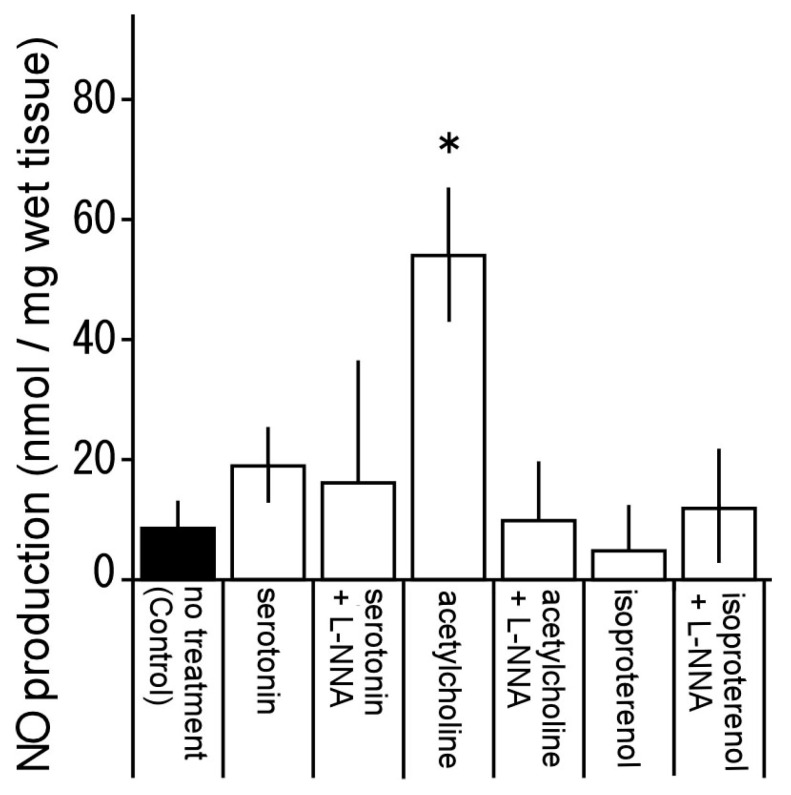
Nitric oxide (NO) production ([NO_2_^−^] + [NO_3_^−^]) induced by serotonin (10^−6^ M), acetylcholine (10^−7^ M), and isoproterenol (10^−6^ M) and the effects of N^ω^-nitro-L-arginine (L-NNA) (10^−4^ M) on the resultant NO production. Control: no treatment. Each column represents the mean ± SEM of arteries from 10 snakes. (* p < 0.05 vs. Control).

To determine whether NO was involved in the relaxation responses induced by serotonin, acetylcholine, and isoproterenol, aorta specimens were treated with serotonin, acetylcholine. and isoproterenol, and NO production was measured (Figure 8). The effect of L-NNA on NO production was also examined; the amounts of NO production induced by serotonin (10^−6^ M) and isoproterenol (10^−6^ M) were not significantly different from those seen in Control (the absence of any treatment). In contrast, a significant increase was observed with acetylcholine (10^−7^ M) versus Control, and the increase in NO production was completely abolished by L-NNA treatment.

### 3.8. Results of Field Observations

We identified a total of 18 individual Tokara habu through observations on the two designated field research dates. Fifteen of the 18 snakes were found in trees (83.3%), at a mean height of 1.3 ± 0.1 m (minimum: 0.5 m, maximum: 1.8 m).

## 4. Discussion

To the best of our knowledge, this is the first report on thoracic aortic responses to noradrenaline, angiotensin II, histamine, rattlesnake bradykinin, isoproterenol, serotonin, and acetylcholine in the Tokara habu. Interestingly, responses to isoproterenol, serotonin, and acetylcholine involved initial relaxation followed by contraction. The response pattern has not been previously reported in other snakes, and in particular, there are no previous reports on aortic relaxation induced by serotonin and isoproterenol in any other snake species.

Tokara habu reportedly exhibit semi-arboreal behavior [11,20], a characteristic substantiated by the findings of our field study. We observed 83.3% (15/18) of the identified snakes in trees. There appear to be some physiological differences between the Tokara habu and the yellow rat snake, despite both species exhibiting similar tree-climbing behavior. Histamine induces no response in the former but causes contraction in the latter [10]. This phenomenon cannot yet be explained, and further studies are required. Multiple previous studies have focused on individual vasoactive substances in a single terrestrial snake species [6,7,8,9], investigating only either contraction or relaxation. However, vascular responses (encompassing both contraction and relaxation) appear more complex for semi-arboreal snakes (the Tokara habu, investigated here, and the yellow rat snake, reported elsewhere [10]) and may involve multiple vasoactive substances.

When compared with terrestrial snakes, the Tokara habu showed a more diverse and stronger relaxation response. The first major difference concerned acetylcholine-induced relaxation, a response that does not occur in terrestrial snakes in the absence of precontraction [8]. Previous studies have shown that the relaxation response usually cannot be observed or is very small without precontraction, for acetylcholine and other agents [18,21,22]. The second major difference concerned the isoproterenol-induced relaxation mediated via β adrenoceptors in the Tokara habu, contrasting with isoproterenol inducing only contraction and not relaxation in a terrestrial snake, the Jararaca [9]. The Tokara habu and Jararaca both belong to the Viperidae family, so we consider that one possible explanation for this physiological difference in vascular response could relate to some adaptation to a semi-arboreal habitat by the Tokara habu.

The Tokara habu may have developed a complex mechanism involving multiple responses to deal with the semi-arboreal nature of its habitat. As a snake moves up or down a tree, its head may be at higher or lower elevations than its heart. Thus, there is a need to adapt to hydrostatic pressure, allowing for flexible changes in blood pressure. The giraffe provides a pertinent example of blood-flow adaptation to gravity. It is known to have physiologically high blood pressure because, under normal blood pressure, blood cannot be pumped to its head, which is at a long distance from the heart and at a considerably higher elevation [23,24], whereas hypertension usually causes a range of disorders in most other animals [25]. Presumably, the Tokara habu and the yellow rat snakes need mechanisms to counteract changes in blood pressure when they climb trees, and we postulate that such mechanisms may involve a relaxation response as part of their blood pressure regulation. However, a single mechanism may not suffice to achieve this regulation. Thus, a variety of relaxation mechanisms could have developed to deal with tree climbing. These mechanisms could include NO-mediated responses, such as those to acetylcholine, and unmediated responses, such as those to isoproterenol and serotonin. Blood pressure and the position of the heart in the body are known to vary in snakes depending on their habitat [2,26,27]. The biphasic thoracic aortic responses to acetylcholine, isoproterenol, and serotonin we noted in the Tokara habu may reflect adaptations to large changes in blood pressure that occur during tree climbing as the snake’s head and heart become vertically aligned.

Focusing on the specific responses induced by vasoactive substances in this study, noradrenaline induced contraction in a concentration-dependent manner, and phentolamine shifted the concentration-response curve for noradrenaline to the right in parallel (Figure 3). The pEC_50_ value (indicating sensitivity for the relevant receptor) for noradrenaline in Tokara habu (6.04) was similar to that in bovine median caudal arteries (6.16), where responses are mediated via α_1_ adrenoceptors [28], as well as to those in other snakes, such as the Indian cobra (*Naja naja*; 6.04) [7], the Jararaca (*Bothrops jararaca*; 6.63) [9], and the yellow rat snake (*Pantherophis obsoletus*; 6.72) [10]. Phentolamine was also reported to have a similar effect in the Jararaca and the Indian cobra [7,9]. These results suggest that noradrenaline-induced contraction in Tokara habu is mediated via α_1_ adrenoceptors.

Amino acid sequencing of angiotensin-like peptides in snakes previously revealed two types of angiotensin II, one identical to the corresponding human peptide and one with a sequence that differs by a single amino acid [29]. Angiotensin II induced contraction in the thoracic aorta of the Tokara habu. In other snakes, angiotensin II also induced aortic contraction [6,7,10]. Renin or renin-like substances are present in the kidneys of many vertebrates, from bony fishes to mammals, and are responsible for blood pressure regulation [30]. These substances may play an important role in blood pressure regulation in snakes. The relevant pEC_50_ values in semi-arboreal snakes, such as the Tokara habu and the yellow rat snake, were 7.39 and 6.50 [10], respectively, and those in terrestrial snakes, such as the Indian cobra and the Jararaca, were 8.73 and 6.76, respectively [6,7]. These figures suggest that sensitivity to the relevant receptor may not be related to habitat.

The β adrenoceptor is classified into β_1_, β_2_, and β_3_ subtypes, all three of which are expressed in smooth muscle [31]. In this study, isoproterenol induced contraction at a high concentration, although it induced relaxation at a low concentration. Propranolol (10^−7^ M) abolished the relaxation response (Figure 4), suggesting that the relaxation had been mediated through β_1_ and/or β_2_ adrenoceptors. This is based on propranolol being a non-selective antagonist of β adrenoceptors, known to block the β_3_ adrenoceptor only at high concentrations (10^−6^ or 10^−5^ M) [31,32,33]. We showed that pretreatment with atenolol and butoxamine significantly curtailed the relaxation response induced by isoproterenol. Therefore, we consider that β_1_ and β_2_ adrenoceptors are present in the thoracic aorta of the Tokara habu. As shown in Figure 8, isoproterenol-treated thoracic aortas did not produce NO, suggesting that the relaxation response induced by isoproterenol is not mediated via NO. Phentolamine shifted the contraction response curve yielded by isoproterenol treatment in the presence of propranolol parallel to the right (Figure 4), suggesting that the contraction was mediated via α adrenoceptors. In another snake species, the Jararaca (a member of the same family as the Tokara habu), isoproterenol induced only contraction, mediated via α adrenoceptors, and not relaxation [9].

Multiple classes of serotonin (5-HT) receptors exist, from 1 to 7, with several additional subtypes [34]. Serotonin reportedly induced basilar arterial contraction via the 5-HT_1_ receptor in the Habu snake [18] and was involved in pulmonary vascular responses (a study with no receptor mediation) in the file snake (*Acrochordus granulatus*) [35]. Many vascular responses to serotonin are constrictive, but reported responses also include relaxation in the rat jugular vein [36,37], pig pulmonary artery [38,39], and equine coronary artery [40]. Although serotonin-induced relaxation responses vary between animal species and blood vessels, 5-HT_1_, 5-HT_2_, and 5-HT_7_ receptors and endothelial cell-derived NO all appear to be involved [34,36,37,38,39,40]. In further experiments with antagonists and inhibitors that effectively yielded serotonin-induced relaxation, the combination of L-NNA, SB269970, methiothepin, or ketanserin inhibited serotonin-induced relaxation at low concentrations but not at high concentrations (Figure 5A). Also, NO production was not observed after treating thoracic aortas with serotonin (Figure 8). These results suggest that serotonin-induced relaxation in the thoracic aorta of the Tokara habu does not involve NO and may involve other relaxing factor(s) in addition to 5-HT_2_ and 5-HT_7_ receptors, but the details are unknown and require further investigation. The contraction induced by serotonin at 10^−5^ M was significantly inhibited by methiothepin, but not by ketanserin (Figure 5B). This result suggests contraction induced via the 5-HT_1_ receptor. 

Both acetylcholine-induced relaxation and contraction responses were significantly inhibited by pirenzepine and pfHHSiD (Figure 6 and Figure 7). In addition, the relaxation response was also abolished by L-NNA, and acetylcholine significantly induced NO production, which was abolished by L-NNA (Figure 6 and Figure 8). These results suggest that the acetylcholine-induced relaxation response was mediated via M_1_ and M_3_ muscarinic receptors, with the production of NO occurring in vascular endothelial cells. Furthermore, as discussed in antecedent sections, the acetylcholine-induced thoracic aortic relaxation response appeared to be stronger in Tokara habu than in other snakes, which may be attributed to vascular responses in other snakes being induced by acetylcholine only after noradrenaline-induced precontraction [8]. To summarize, in this study, acetylcholine induced relaxation mediated by NO via M_1_ and M_3_ receptors in vascular endothelial cells at a low concentration, and induced contraction mediated via M_1_ and M_3_ receptors in vascular smooth muscle at a high concentration.

Histamine induces strong tachycardia elicited via H_1_ and H_2_ receptors under anesthesia [41,42]. However, histamine may not be involved in the vascular response in Tokara habu thoracic aortas.

Bradykinin has previously been reported to show amino acid sequences that differ between humans and mammals at only two positions. In snakes, its vasoactive effect reportedly induces relaxation, mediated by NO or an endothelium-derived relaxing factor [18,43]. In this study, rattlesnake bradykinin had no effect on resting tension or precontraction. Therefore, we consider that rattlesnake bradykinin may not be involved in the thoracic aortic vasoreactivity in the Tokara habu.

## 5. Conclusions

In the thoracic aorta of the Tokara habu, noradrenaline and angiotensin II induced contraction, whereas acetylcholine, isoproterenol, and serotonin induced relaxation followed by contraction. Histamine and rattlesnake bradykinin had no effect on resting tension. Unlike the monotonous vasoreactivity of terrestrial snakes, vasoreactivity in the Tokara habu may be governed by diverse regulatory mechanisms that are characteristic of snakes apparently adapted to their semi-arboreal habitat.

## Figures and Tables

**Table 1:** pEC_50_ values and maximal response (E_max_) to agonists.

Agonists	pEC_50_	E_max_ (%) (Reactivity)
Resting condition (2.4 mN)		
Noradrenaline	6.04 ± 0.14	130.2 ± 13.0 (contraction ^a^)
Angiotensin II	7.39 ± 0.06	46.4 ± 10.3 (contraction ^a^)
Histamine	−	0 (no response)
Rattlesnake bradykinin	−	0 (no response)
Isoproterenol	7.11 ± 0.07	22.9 ± 0.3 (relaxation ^b^)
Serotonin	7.41 ± 0.28	27.6 ± 4.9 (relaxation ^b^)
Acetylcholine	8.25 ± 0.19	10.2 ± 3.1 (relaxation ^b^)

pEC_50_: negative logarithm of half maximal effective concentration (EC_50_), E_max_: the maximum effect. ^a^ Contraction induced by 60 mM KCl was taken as 100%. ^b^ Relaxation induced by sodium nitroprusside (10^−4^ M) was taken as 100%.

## Data Availability

The data presented in this study are available on request from the corresponding author.
